# Enhancement of Hypoxia Tolerance of Gibel Carp (*Carassius auratus gibelio*) via a Ferroporphyrin-Rich Diet

**DOI:** 10.3390/antiox14060738

**Published:** 2025-06-16

**Authors:** Hualiang Liang, Haifeng Mi, Kai Wang, Mingchun Ren, Lu Zhang, Dongyu Huang, Jiaze Gu

**Affiliations:** 1 Key Laboratory of Integrated Rice-Fish Farming Ecology, Ministry of Agriculture and Rural Affairs, Freshwater Fisheries Research Center, Chinese Academy of Fishery Sciences, Wuxi 214081, China; lianghualiang@ffrc.cn (H.L.); huangdongyu@ffrc.cn (D.H.); 2 Healthy Aquaculture Key Laboratory of Sichuan Province, Tongwei Co., Ltd., 588 Tianfu Avenue, Chengdu 610093, China; mihf@tongwei.com; 3 College of Fisheries and Life of Science, Shanghai Ocean University, Shanghai 201306, China; 18809866393@163.com; 4 Wuxi Fisheries College, Nanjing Agricultural University, Wuxi 214081, China; 2024213006@stu.njau.edu.cn

**Keywords:** gibel carp (*Carassius auratus gibelio*), ferroporphyrin, hypoxia stress, metabolism, oxygen transport, antioxidant capacity, diet trial

## Abstract

Gibel carp (*Carassius auratus gibelio*) were hypoxia stressed for 12 h after an 8-week FPR nutrient-enriched feeding experiment, which was to evaluate the role of FPR in hypoxic stress in gibel carp (*Carassius auratus gibelio*). The dissolved oxygen was reduced to a range of 0.6 ± 0.2 mg/L. Results showed that FPR supplementation could maintain the osmotic pressure equilibrium by improving the ion concentrations of plasma including Na^+^, Ca^+^ and K^+^, and Na^+^/K^+^-ATPase activity of liver. FPR supplementation could effectively enhance the antioxidant capacity by improving the levels of GPX, SOD, CAT, and GSH, and reduce the level of MDA. FPR supplementation could improve the core gene expressions of Nrf2 signalling pathway including *nrf2*, *sod*, *ho-1*, *gpx,* and *cat*. The high levels of FPR supplementation (0.04%) might had a negative effect on immunity. FPR supplementation could improve the expression levels of HIF-1 signalling pathway-related genes to adapt to hypoxia condition including *hif-1α*, *epo*, *angpt1*, *vegf*, *et1*, and *tfr-1*. These results also were supported by higher SR and number of gill mitochondria in FPR supplementation. In general, the appropriate FPR supplementation was 0.01% based on the results of this study and economic cost, which could heighten hypoxic adaptation and SR.

## 1. Introduction

Dissolved oxygen (DO), a crucial factor in aquatic environments, is easily influenced by climate variations and physical and chemical factors [1]. In recent years, due to global climate variations and the expansion of human activities, hypoxia duration has been prolonged, and hypoxia conditions have become more frequent [2]. This change is more likely to occur in aquaculture due to high stocking density and overfeeding [3]. Furthermore, previous research indicated that hypoxia represents a significant factor contributing to the natural mortality of fish in natural aquatic environments during the summer and winter seasons [4]. Consequently, hypoxia is becoming increasingly prevalent in aquaculture processes and natural aquatic ecosystems [5]. In aquaculture, hypoxia stress can inhibit growth, immunocompetence, antioxidant, and metabolic patterns of cultured fish. In severe cases, this can lower survival rate (SR), which can lead to significant economic losses [6,7]. In order to adapt to hypoxia conditions, fish engage in a complex physiological process, which includes enhancing oxygen-carrying capacity, regulating mitochondrial function, maintaining cytoprotective strategies (such as osmotic pressure and antioxidant system), and inhibiting metabolism [8,9]. A significant number of previous studies have found that the modulation of fish physiological processes by nutritional means can effectively improve their hypoxia tolerance [10,11].

The addition of additives to diet is currently regarded as an effective means of enhancing hypoxia tolerance in aquatic animals [11,12,13]. Ferroporphyrin (FPR) and iron (III) protoporphyrinium chloride (IX), represents the in vitro form of haemoglobin [1]. It is widely distributed in nature and is extracted primarily from the globular linkages of haemoglobin in animals [14]. FPR plays a number of physiological roles, including the participation in oxygen transport, promotion of erythropoiesis and maturation, and scavenging of hydrogen peroxide from the body [15]. Furthermore, FPR serves as a substrate for the heme oxygenase (HO) enzyme and functions as an inducer and activator of heme oxygenase-1 (HO-1) [16], which is a microsomal enzyme that is responsive to stimuli associated with oxidative stress and inflammation [17]. It facilitates the synthesis of CO, iron, and biliverdin from heme, and biliverdin can undergo further reduction to yield bilirubin, then CO and bilirubin elicit cytoprotective, immunomodulatory, antioxidant, and anti-inflammatory responses, and free iron enhances the expression of ferritin, which mitigates the deleterious effects of oxidative stress [18]. Previous research has demonstrated that FPR attenuates obesity-induced oxidative stress and inflammation in mice [19] and modulates the immune response to alleviate influenza pneumonia [20]. Furthermore, FPR may indirectly inhibit the HIF-1 signalling pathway by directly blocking heat shock protein 90 [21]. Nevertheless, the precise manner by which FPR regulates the hypoxia defence mechanism in fish remains unclear.

The HIF signalling pathway represents a vital cellular system in the maintenance of oxygen homeostasis within cells [22], and hypoxia stress in fish is subject to regulation by the HIF signalling pathway [23]. HIF-1 is a major regulator of the hypoxia signalling pathway, which binds to hypoxia-responsive components and induces a cascade of responses to hypoxia, including erythropoiesis, angiogenesis, and apoptosis [24]. The HIF-1 complex comprises two main components, namely, the hypoxia-inducible factor-1α (HIF-1α) and the aryl hydrocarbon receptor nuclear transport protein [11]. The availability of HIF-1 is primarily contingent upon the presence of HIF-1α [9]. HIF-1α is among the earliest response factors by which organisms detect hypoxia and is regarded as a susceptibility factor for hypoxia [25]. In condition of normal hypoxia, HIF-1α is degraded via the proteasome pathway, thereby maintaining low expression levels. However, the proteasomal degradation pathway is inhibited under hypoxia conditions, resulting in increased *hif-1α* expression levels [26,27,28]. It has been established through previous studies that significantly higher *hif-1α* expression levels were found in the livers of Atlantic croaker (*Micropogonias undulatus*) and Pacific bluefin tuna (*Thunnus orientalis*) when exposed to hypoxia conditions [29,30]. Moreover, the *hif-1α* expression was regulated by nuclear factor kappa-B (NF-κB) signalling pathway [23]. However, in gibel carp (*Carassius auratus gibelio*), the effect of FPR on hypoxia stress in HIF-1 signalling pathway and related NF-κB signalling pathway has not been reported yet. In addition, hypoxia induces oxidative stress thereby promoting cellular reactive oxygen species (ROS) production [31]. Excessive ROS can disrupt the body’s oxidative system, thereby causing oxidative damage to the body, which is regulated through the nuclear factor erythroid 2-related factor 2 (Nrf2) signalling pathway in this physiological process of oxidative stress [32].

The gibel carp is an economically significant freshwater fish in China, with an annual production of up to 2.84 million tonnes in 2023 [33]. With aquaculture scale expansion and aquaculture density increasing, hypoxia has become one of the most common causes in the domain of aquaculture. In previous studies of gibel carp, the hypoxia conditions could lead to some negative effects, such as inhibiting growth performance, inducing oxidative stress, and structural damage [34,35], which is one of the crucial limiting factors in aquaculture production of gibel carp. Currently, there is little research on the ability of FPR to hypoxia resistance in gibel carp. The study was to ascertain whether the incorporation of FPR into the diet could enhance the hypoxia tolerance by influencing these mechanisms.

## 2. Materials and Methods

### 2.1. Hypoxia Stress Challenge

The basal diet of isoenergetic and isonitrogenous were designed based on commercial formulation principles, presented in our previous study [36]. Five experimental groups were designed by adding five levels of FPR to the basic formula based on the recommended usage amount of the product from the company, which were 0 mg/kg, 100 mg/kg, 200 mg/kg, 300 mg/kg and 400 mg/kg, respectively. The type of FPR we use is food-grade heme iron (hemin 97%, iron 2%), which cost $55/kg. Firstly, an 8-week FPR nutrient-enriched feeding experiment was carried out. During a previous feeding trial, the water temperature was maintained at 29 ± 1 °C (Thermometers, Shanghai Sundragon Electric Appliance Co., Ltd., Shanghai, China), the dissolved oxygen concentration was maintained at ≥6.4 mg/L (Pen Dissolved Oxygen Metre, Dongguan Wanchuang Electronic Products Co., Ltd., Guangzhou, China), and the pH exhibited fluctuations between 7.65 ± 0.35 (Dr Water kit, Heachen Energy Technology Co., Ltd., Shanghai, China). The principle of apparent satiety feeding was adopted in the previous feeding trial. After collecting the samples of our previous study [36], the remaining fourteen fish (87.55 ± 0.96 g) from each cage were transferred to recirculating water tanks with a volume of 300 L for 12 h of hypoxia stress. The dissolved oxygen was reduced to a range of 0.6 ± 0.2 mg/L (Pen Dissolved Oxygen Metre, Dongguan Wanchuang Electronic Products Co., Ltd., Guangzhou, China) through the use of nitrogen aeration, and the water temperature was maintained in the range of 20.1 ± 0.6 °C (Thermometers, Shanghai Sundragon Electric Appliance Co., Ltd., Shanghai, China). These parameters were measured every 3 h. Subsequently, the mortality number of fish in each tank was recorded (Table S1).

### 2.2. Sample Collection

After 12 h of hypoxia stress, the three surviving fish from every tank were subjected to sampling of their blood, gills, and livers. Firstly, MS-222 (100 mg/L) was used for anaesthetizing sample fish. Blood samples were collected from the tail vein of fish, which aimed to collected plasma by centrifuge (5000 rpm at 4 °C, 10 min). Then fish were dissected to obtain the livers and gills. The liver was frozen in liquid nitrogen and kept at −80 ° C for further analysis. The gills were preserved using glutaraldehyde fixative for subsequent transmission electron microscopy analysis.

### 2.3. Transmission Electron Microscopy Analysis

Fresh gill samples measuring 1 mm^3^ were collected and preserved in EP tubes with glutaraldehyde fixative. Then the fixed tissue samples were rinsed using 0.1 M phosphate buffer PB (pH 7.4) on three occasions, with each rinse lasting 15 min. Subsequently, the samples were sequentially dehydrated using a series of alcohol solutions with concentrations ranging from 30% to 100% for a duration of 20 min at each concentration. This process was repeated with 100% acetone twice, with each acetone treatment lasting for 15 min. Following this, osmotic embedding was performed through a mixture of acetone and embedding agent. Subsequently, polymerisation was carried out, and the embedding plate was placed in an oven at 60 °C for 48 h. Thereafter, the resin block was extracted and set aside. The resin block was sliced into 1.5 μm semi-thin sections on a semi-thin sectioning machine, stained with toluidine blue and positioned under a light microscope. The resin block was then sliced using an ultra-thin sectioning machine (60–80 nm), with 150-mesh copper mesh being utilised for this purpose. The copper mesh was subjected to staining with a 2% uranyl acetate saturated alcohol solution for a period of 8 min. This was followed by washing with 70% alcohol, which was repeated three times. The copper mesh was then washed with ultrapure water, a process which was also repeated three times. Next, the copper mesh was stained with 2.6% lead citrate in a carbon dioxide solution for 8 min. This was followed by washing with ultrapure water, which was repeated three times. Finally, the sections were observed under a transmission electron microscope and the images were collected for analysis.

### 2.4. Biochemical Analysis

The kits purchased from Nanjing Jiancheng Bioengineering Institute (Nanjing, China) were used to test the activities of hepatic related enzymes. Following the operating instructions, the approaches are described as below. Glutathione peroxidase (GPX) was measured by the colorimetric method using the model A005-1-2 kit, the principle is that GPX can promote the reaction between hydrogen peroxide (H_2_O_2_) and reduced glutathione (GSH) to produce H_2_O and oxidised glutathione (GSSG), the activity of GPX can be expressed by the speed of the enzyme reaction, and the activity of the enzyme can be determined by measuring the consumption of reduced glutathione in the enzyme reaction. The sodium potassium pump (Na^+^/K^+^-ATPase) was measured by the microplate method using the model A070-2-2 kit, the principle is that ATPase breaks down ATP to produce ADP and inorganic phosphorus, and the amount of inorganic phosphorus can be measured to determine the level of ATPase activity. The superoxide dismutase (SOD) was measured by the WST-1 method using the model A001-3-2 kit, the meaning of the result is that one unit of SOD activity (U) is the amount of enzyme required to inhibit 50% of the oxidation rate per reactive solution and per milligram of protein in 1 mL of reactive solution. The catalase (CAT) was measured by the visible light method using the model A007-1-1 kit, the principle is that the reaction of cat decomposition of H_2_O_2_ can be rapidly aborted by adding ammonium molybdate, and the remaining H_2_O_2_ interacts with ammonium molybdate to produce a yellowish complex, which can be measured at 405 nm to calculate the CAT activity. About the contents of hepatic antioxidant-related indexes, malondialdehyde (MDA) was measured by the TBA method using the model A003-1-2 kit, the principle is that MDA can condense with TBA to form a red product with a maximum absorption peak at 532 nm. The GSH was measured by the microplate method using the model A006-2-1 kit, the principle is that GSH can react with dithiodinitrobenzoic acid (DTNB) to produce a yellow compound, which can be colorimetrically quantified at 405 nm to determine the GSH content. For the ion concentrations of plasma, Na^+^ was measured by the colorimetric method using the model C002-1-1 kit, the principle is that Na^+^ and potassium antimony 6-hydroxide form a homogeneous turbidity with Na^+^ in the presence of a dispersant and a remover, absorbance values were measured at 620 or 630 nm to calculate the content. The Cl^−^ was measured by the microplate method using the model C003-2-1 kit, the principle is to treat Cl^−^ with mercury thiocyanate to form coloured complexes whose depth of colour is proportional to the concentration of Cl^−^. The Ca^2+^ was measured by the microplate method using the model C004-2-1 kit, the principle is that Ca^2+^ in the sample combines with methylthymol blue (MTB) in an alkaline solution to produce a blue complex; the amount of calcium in the sample can be calculated by comparing the colorimetry with a calcium standard of the same treatment; and K^+^ was measured by the turbidimetry assay using the model C001-1-1 kit, the principle is that in an alkaline medium, K^+^ in serum samples treated with a protein precipitant reacts with NA-TPB to produce turbidity and a stable suspension, the turbidity is proportional to the concentration of K^+^ in the sample. The resultant data were read on a Spectrophotometer (Thermo Fisher Multiskan GO, Shanghai, China), after which calculations were performed according to the corresponding kits instructions. Other details of the determination are shown in Table 1 and our previous study [36].

### 2.5. Genes Expression Level Analysis of Liver

The total RNA of liver was extracted by RNAiso plus kit of Vazyme Biotech Co., Ltd. (Nanjing, China), the concentration and quality of total RNA were assessed using Nano Drop 2000 spectrophotometer of Thermo Fisher Multiskan GO (Shanghai, China), and the concentrations of the RNA solutions were diluted to 60 ng/μL, and the index of A260/A280 were between 1.8 and 2.0, indicating that the RNA purity met the requirement. Real-time PCR (RT-PCR) was performed using the One Step qRT-PCR SYBR Green Kit of Vazyme Biotech Co., Ltd. (Nanjing, China) on a CFX96 Touch of Bio-Rad (Hercules, CA, USA). The used primer sequences designed for the experiments were listed in Table 2. *β-actin* was chosen as the control gene for its high and stable expression, which did not significantly differ among different treatments. The expression levels of related genes were determined by the relative standard curve method.

### 2.6. Data Processing

The SPSS 26.0 was conducted to analysis data. The one-way ANOVA (Tukey’s test) was also conducted to clarify the differences between groups. Results were presented with the means ± SE. Three biological replicates in each group. The value of *p* < 0.05 were considered to be significant differences.

## 3. Results

### 3.1. The Ion Concentrations of Plasma and the Activity of Na^+^/K^+^-ATPase of Liver After Hypoxia Stress

Table 3 presents the ion concentrations of plasma and Na^+^/K^+^-ATPase activity of liver following a 12 h hypoxia stress. Compared with the control group, the 0.01% and 0.02% FPR supplementation significantly improved the activity of Na^+^/K^+^-ATPase in liver and concentration of Na^+^ in plasma, respectively (*p* < 0.05). The 0.02% FPR supplementation significantly lowered the concentration of K^+^ (*p* < 0.05). Concurrently, the significantly decreased concentrations of Ca^+^ were presented in 0.02% and 0.03% FPR supplementation (*p* < 0.05). Furthermore, the concentration of Cl^−^ was not found to change significantly in the FPR supplementation (*p* > 0.05).

### 3.2. Antioxidant-Related Parameters in Liver After Hypoxia Stress

**Figure 1:**
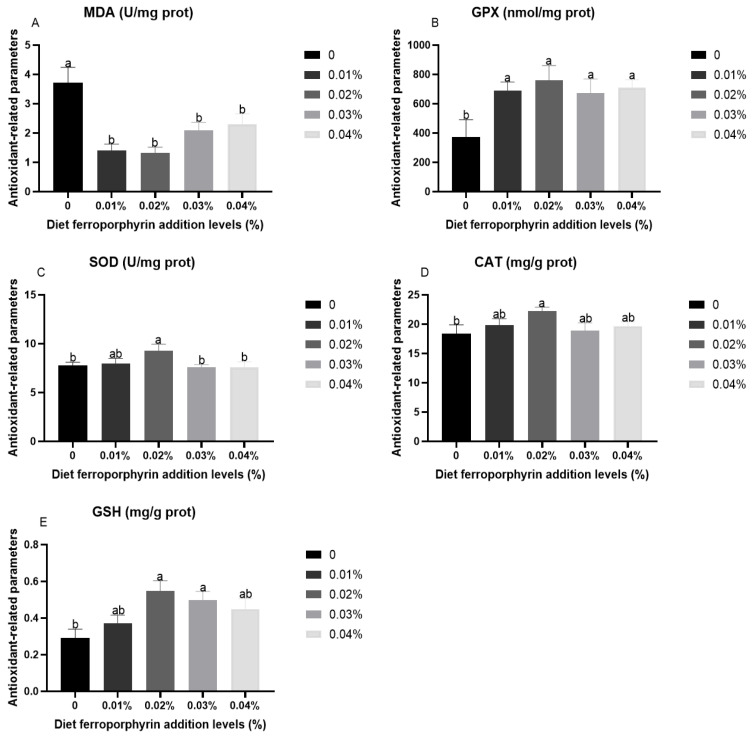
Antioxidant-related parameters in liver after hypoxia stress: (**A**) MDA; (**B**) GPX; (**C**) SOD; (**D**) CAT; (**E**) GSH. Results are presented with the means ± SE. (n = 3). Results with different superscript letters (a, b) represent significant differences (*p* < 0.05).

Figure 1 presents the results of antioxidant-related parameters in gibel carp after hypoxia stress. Compared with the control group, significantly lower levels of MDA were presented in the FPR supplementation (*p* < 0.05). Conversely, the FPR supplementation was significantly higher in the activities of GPX (*p* < 0.05). The significantly high activities of CAT and SOD were presented in 0.02% FPR supplementation (*p* < 0.05). The 0.02% and 0.03% FPR supplementation presented a significant improvement in the contents of GSH (*p* < 0.05).

### 3.3. The Expression Levels of Antioxidant-Related Genes in Liver After Hypoxia Stress

**Figure 2:**
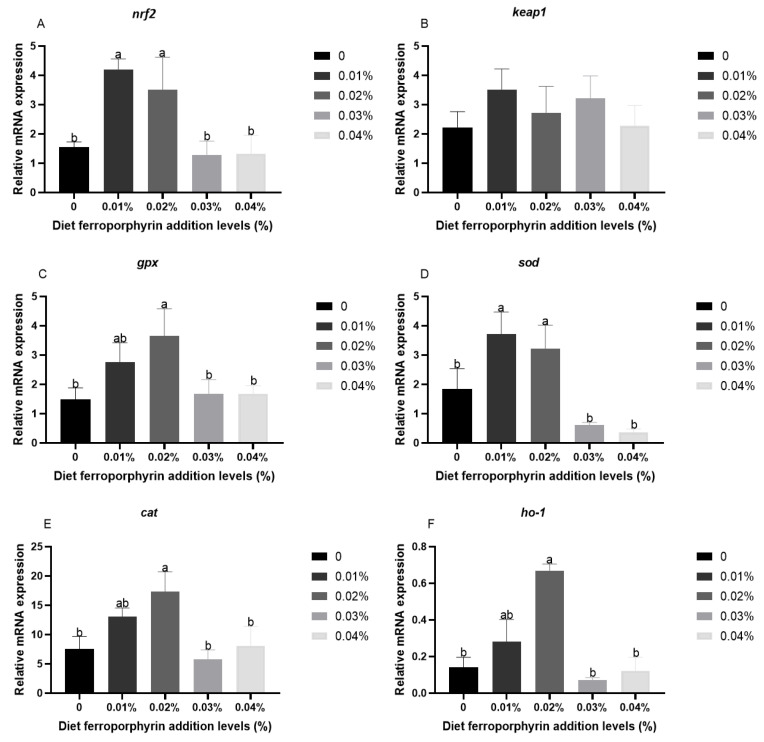
The response of antioxidant-related genes after hypoxia stress: (**A**) *nrf2*; (**B**) *keap1*; (**C**) *gpx*; (**D**) *sod*; (**E**) *cat*; (**F**) *ho-1*. Results are presented with the means ± SE. (n = 3). Results with different superscript letters (a, b) represent significant differences (*p* < 0.05).

Figure 2 presents the expression levels of antioxidant-related genes in liver after hypoxia stress. Compared with the control group, the 0.01 and 0.02% FPR supplementation presented a significant improvement in expression levels of *nrf2* and *sod* (*p* < 0.05). The significantly high expression levels of *ho-1*, *gpx,* and *cat* were presented in the 0.02% FPR supplementation (*p* < 0.05). Furthermore, the FPR supplementation had no effect on the expression level of *keap1* (*p* < 0.05).

### 3.4. The Expression Levels of NF-κB Signalling Pathway-Related Genes in Liver After Hypoxia Stress

**Figure 3:**
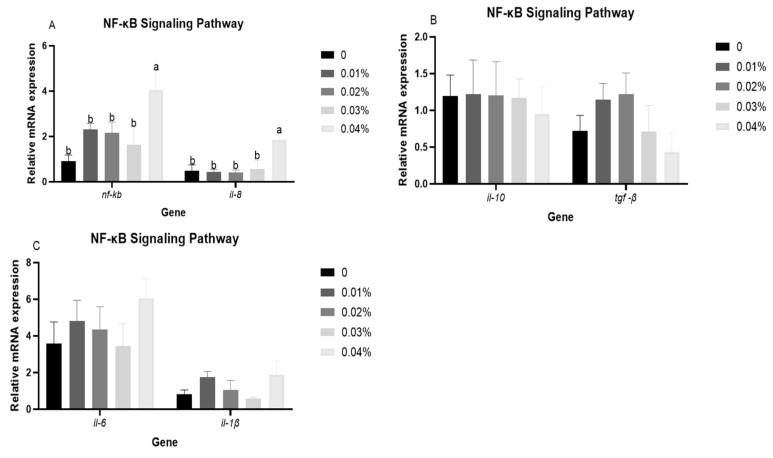
The response of NF-κB signalling pathway-related genes after hypoxia stress: (**A**) *nf-kb* and *il-8*; (**B**) *il-10* and *tgf-β*; (**C**) *il-1β* and *il-6*. Results are presented with the means ± SE. (n = 3). Results with different superscript letters (a, b) represent significant differences (*p* < 0.05).

Figure 3 presents the expression levels of NF-κB signalling pathway-related genes in liver after hypoxia stress. Compared with the control group, significant higher expression levels of *il-8* and *nf-kb* were shown in the 0.04% FPR supplementation (*p* < 0.05). Furthermore, the expression levels of *il-1β*, *tgf-β*, *il-6*, and *il-10* were not affected by FPR supplementation (*p* > 0.05).

### 3.5. The Expression Levels of HIF-1 Signalling Pathway-Related Genes in Liver After Hypoxia Stress

**Figure 4:**
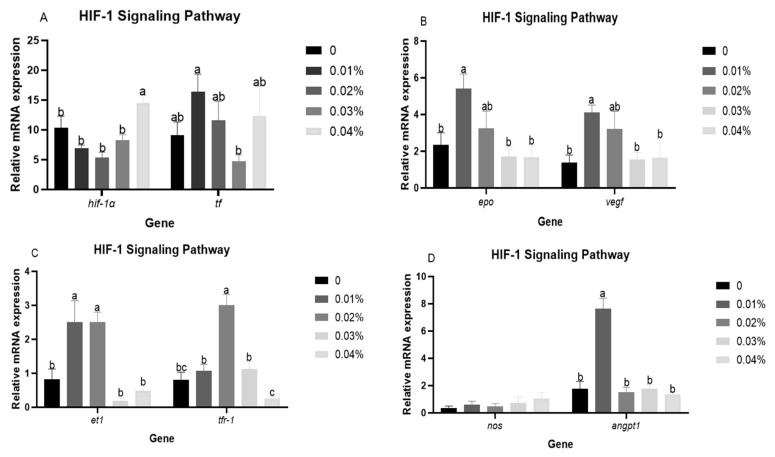
The response of HIF-1 signalling pathway-related genes after hypoxia stress: (**A**) *hif-1α* and *tf*; (**B**) *epo* and *veg*f; (**C**) *et1* and *tfr-*1; (**D**) *nos* and *angpt1*. Results are presented with the means ± SE. (n = 3). Results with different superscript letters (a, b, c) represent significant differences (*p* < 0.05).

Figure 4 presents the expression levels of HIF-1 signalling pathway-related genes after hypoxia stress. The FPR supplementation resulted in a decreasing trend and then increasing expression levels of *hif-1α*. The highest expression level of *hif-1*α was presented in the 0.04% FPR supplementation (*p* < 0.05). The 0.01% FPR supplementation presented a significant improvement in expression levels of *epo*, *angpt1*, and *vegf* (*p* < 0.05). Significant higher expression levels of *et1* were presented in the 0.01% and 0.02% FPR supplementation (*p*< 0.05). The expression level of *tfr-*1 was increased and then decreased, reaching a maximum in the 0.02% FPR supplementation (*p* < 0.05). In addition, the expression levels of *tf* and *nos* were not affected by FPR supplementation (*p* > 0.05).

### 3.6. The SR of Gibel Carp and Number of Mitochondria in Gill After Hypoxia Stress

**Figure 5:**
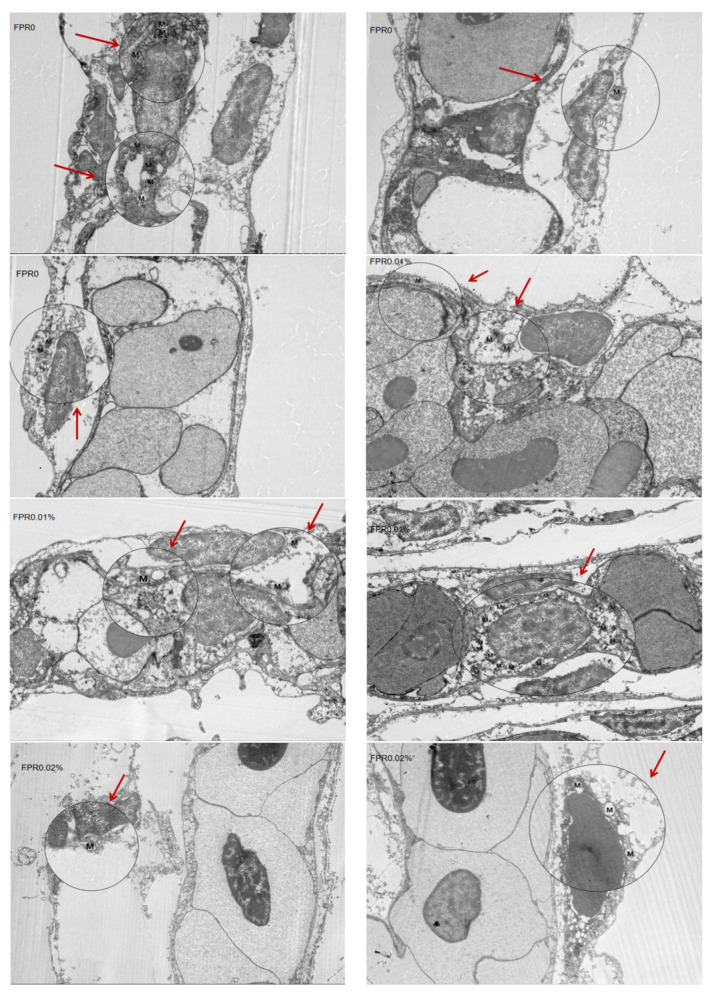

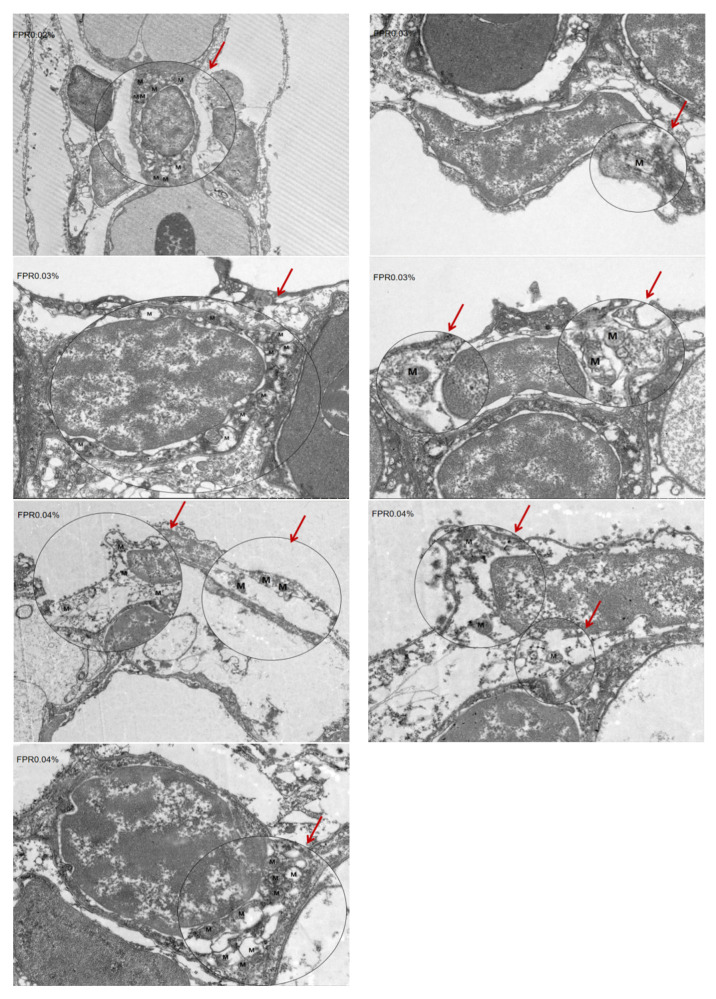
The number of mitochondria in gills (an M in the picture represented a mitochondrion).

Figure 5 and Table 4 present the SR of gibel carp and number of mitochondria in gills after hypoxia stress. The mean number of mitochondria in gills was 3.3 in the control group. In addition, the FPR supplementation exhibited the mean number of mitochondria (*p* > 0.05). The control group exhibited a lowest SR compared with other groups (*p* < 0.05), and the FPR supplementation presented a significant improvement in SR (*p* < 0.05).

## 4. Discussion

Oxygen is a vital component for aquatic animals; however, indirect or chronic hypoxia often leads to a reduction in feed intake, slower growth, and increased disease prevalence and mortality in cultured fish [41]. Substantial evidence indicates that the SR of fish is decreased markedly in acute hypoxia [42]. Furthermore, the SR serves as a visual indicator of the fish species’ tolerance to hypoxia. In the present study, the SR of the group without FPR supplementation only has 23.81%. By contrast, the group received supplementation with 0.01–0.04% FPR exhibited a significantly higher SR. This is similar to the results of previous studies, showing that vitamin C (VC) plus taurine supplementation improved survival in gibel carp [43]. These results indicated that FPR supplementation enhances the gibel carp’s capacity to withstand hypoxia condition.

Hypoxia impairs electron transfer efficiency within the respiratory chain of aquatic animals, consequently leading to the generation of deleterious ROS, which cause oxidative stress [44]. The antioxidant defence system represents a crucial response mechanism for fish to mitigate oxidative stress, which encompasses three pivotal enzymes, namely SOD, GPX, and CAT, which operate in a synergistic capacity to attenuate the deleterious effects of free radicals on tissue cells [45,46]. Previous studies have demonstrated that the activity of antioxidant enzymes was diminished in aquatic animals subjected to acute stress [47]. This suggests that the antioxidant defence system of aquatic animals is unable to effectively eliminate the damage caused by peroxidation products [46]. In this experiment, the FPR supplementation increased the activities of CAT, GPX, and SOD, which was similar to the study that the VC supplementation in the diet of gibel carp increased the activities of CAT, GPX, and SOD in response to acute hypoxia condition [32]. Moreover, the 0.02% and 0.03% FPR supplementation could increase the GSH contents, which is a molecule with the capacity to regulate redox reactions by eliminating surplus ROS, thereby safeguarding cells from oxidative stress [48]. It has been demonstrated that hypoxia stress resulted in oxidative damage and the abundant formation of peroxidation products including MDA, which is indicative of the degree of oxidative damage sustained by fish tissue cells [49,50]. This study found that the FPR supplementation was able to significantly reduce MDA levels. The above results indicated that the FPR supplementation could effectively enhance the antioxidant capacity of gibel carp, thereby eliminating ROS generated by hypoxia stress and safeguarding gibel carp from free radicals. This outcome is also aligned with the observed SR of this study. These results indicated that the FPR supplementation had the positive effect on the Nrf2/Keap1 signalling pathway, which is identified as the most crucial endogenous antioxidant signalling pathway in animals [51]. The 0.01% and 0.02% FPR supplementation has been shown to significantly improve the expression levels of *nrf2*. In addition, the same level of FPR supplementation markedly elevated the expression levels of *gpx*, *cat*, and sod. HO-1 is also a downstream gene of Nrf2 and represents a crucial defence mechanism for cellular responses to oxidative stress [52]. The 0.02% FPR supplementation was found to be a significant improvement in the expression level of *ho-1*. These results further supported that the FPR supplementation in diet could effectively improve the hepatic antioxidant capacity in gibel carp, thereby improving the adaptability of gibel carp to hypoxia stress. One potential explanation for this outcome is that the HO-1 facilitates the oxidative degradation of FPR, yielding carbon monoxide, biliverdin, and iron. Subsequently, carbon monoxide induces Nrf2, which in turn activates antioxidant response elements, thereby regulating the expression of multiple antioxidant enzyme-related genes [53].

A close relationship has been demonstrated between the hypoxia response and the immune response [54]. Hypoxia stress affects the immune system of fish by inducing an inflammatory response in cells [55]. In this study, the 0.04% FPR supplementation could improve the expression level of *nf-kb*, which was similar to the result that diet with taurine and VC in gibel carp subjected to hypoxia stress [43]. Meanwhile, the expression levels of *il-8* showed the same trend as *nf-kb*. These results indicated that the ingestion of higher levels of FPR might generate inflammatory response under hypoxia condition. Our previous study also found that the high levels of FPR supplementation resulted in the impairment of immune function under normoxic conditions [36]. The HIF signalling pathway represents a principal signalling pathway in organisms in response to hypoxia stress, which is involved in regulating the transcription of a number of factors, including erythropoietic factors, angiogenic factors, ferritin and endothelin [56,57]. In the present study, FPR supplementation influenced both HIF-1 signalling pathways, showing that the 0.04% FRP supplementation presented a significant improvement in the expression level of *hif-1α*. The elevated expression level of *hif-1α* might be due to the fact that the increased FPR supplementation improved the expression level of *nf-kb*, and the *nf-kb* subunits p50 and p65 could bind at the *hif-1α* promoter, which in turn enhanced the transcription of *hif-1α* to increase *hif-1α* expression [23]. Previous studies also found the regulation of *hif-1α* by *nf-κb* expression under hypoxia condition [58,59,60], which was consistent with present results. In response to hypoxia, the body activates defensive mechanisms such as *epo*, *vegf*, and *angpt1*, which promote gas exchange and oxygen transport [61,62]. In this study, the 0.01% FPR supplementation improved expression levels of *epo*, *et1*, *vegf*, and *angpt1*, which indicated that a moderate quantity of FPR supplementation might facilitate erythropoiesis and angiogenesis, thereby augmenting the oxygen-carrying and oxygen-transport capability of gibel carp in response to hypoxia stress. Furthermore, the 0.02% FPR supplementation also improved the expression level of *tfr-1*, a pivotal regulator of cellular iron uptake, playing a crucial role in erythropoiesis [63]. The above results suggested that the FPR may facilitate the body’s capacity to transport and deliver oxygen by modulating the HIF-1 signalling pathway, thus enhancing the body’s ability to adapt to hypoxia conditions.

Energy is a fundamental requirement for the sustenance of life. Aerobic metabolism represents a crucial mechanism for energy acquisition in the majority of organisms [64]. In the conditions of hypoxia, the process of aerobic metabolism is inhibited, which results in a reduction in energy production [65]. Mitochondria is the centre of cellular aerobic metabolism and ATP production, as well as one of the main organelles that regulate hypoxia stress in organisms [66,67], which are sensitive to hypoxia environments and demonstrate a high level of sensitivity to such conditions. Previous studies have demonstrated that hypoxia stimulation significantly compromised mitochondrial function, leading to alterations in mitochondrial morphology and number, depolarisation of the mitochondrial membrane, diminished ATP production, and calcium ion overload [68]. Furthermore, the number of mitochondria is indicative of the level of cellular energy metabolism activity [69]. The present study demonstrated that the 0.01–0.04% FPR supplementation could increase mitochondrial number in gills, which was consistent with previous study [43]. The role of Na^+^/K^+^-ATPase regulates ion transport and energy metabolism [70]. The enzyme consumes energy through active transport, excretes Na^+^ and absorbs K^+^, thereby maintaining intra- and extracellular ion gradients and electrostatic membrane homeostasis [71,72]. Previous studies have demonstrated that hypoxia impaired Na^+^/K^+^-ATPase activity in gibel carp, resulting in disrupted ion regulation [31]. In this study, the 0.01 and 0.02% FPR supplementation enhanced the activities of hepatic Na^+^/K^+^-ATPase in gibel carp. Concomitantly, the 0.02% FPR supplementation improved the level of Na^+^ and decreased the level of K^+^ in plasma, and the level of Ca^+^ presented a negative correlation with the level of Na^+^ in this study. The results showed that FPR supplementation in diet promoted the intracellular metabolism of energy, thereby maintaining the normal physiological activity of the organism. This result might be due to mitochondria undergoing autophagy under hypoxia condition, resulting in the formation of ROS, and the formation of ROS by mitochondria can enhance Na^+^/K^+^-ATPase degradation through the ubiquitin coupling system [73,74]. The FPR supplementation effectively increased the antioxidant capacity of gibel carp, thereby preventing mitochondrial autophagy and reducing ROS, which prevented the degradation of Na^+^/K^+^-ATPase. In general, the aforementioned results indicated that the appropriate levels of FPR supplementation (0.01% and 0.02%) in diet could maintain the osmotic pressure equilibrium of gibel carp and enhance its antioxidant and oxygen transport capability, thereby facilitating the maintenance of normal physiological processes within the organism in hypoxia environments.

## 5. Conclusions

The appropriate FPR supplementation was 0.01% based on the results of this study and economic costs, which improved antioxidant capacity and cellular energy metabolism, stimulated angiogenesis, oxygen transport, and number of gill mitochondria, thereby improving the SR of gibel carp under hypoxia stress. However, the high levels of FPR supplementation (0.04%) might have had a negative effect on immunity in gibel carp by activating the NF-κB signalling pathway. Based on our conclusion, an appropriate level of FPR supplementation was 0.01% in diet, and the cost of added FPR per ton of feed was about $5.5.

## Figures and Tables

**Table 1:** The chemical analysis used in this study.

Items	Methods	The Model of Commercial Kits	Tested Wavelength	Testing Equipment/Assay Kits
CAT	Visible light method	Model A007-1-1	405 nm	Assay kits from Nanjing Jiancheng Bioengineering Institute (Nanjing, China); Spectrophotometer (Thermo Fisher Multiskan GO, Shanghai, China).
GSH	Microplate method	Model A006-2-1	405 nm	
SOD	WST-1 method	Model A001-3-2	450 nm	
MDA	TBA method	Model A003-1-2	532 nm	
GPX	Colorimetric method	Model A005-1-2	412 nm	
Na^+^/K^+^-ATPase	Microplate method	Model A070-2-2	636 nm	
Na^+^	Colorimetric method	Model C002-1-1	620 nm	
Cl^−^	Microplate method	Model C003-2-1	505 nm	
Ca^2+^	Microplate method	Model C004-2-1	610 nm	
K^+^	Turbidimetry assay	Model C001-1-1	440 nm	

**Table 2:** Primer sequences for this study.

Gene	Forward Primer (5′-3′)	Reverse Primer (5′-3′)	Accession Number/Reference
*β-actin*	GATGATGAAATTGCCGCACTG	ACCGACCATGACGCCCTGATGT	[37]
*keap1*	CTCCGCTGAATGCTACAA	GGTCATAACACTCCACACT	XM_026245355.1
*nrf2*	TACCAAAGACAAGCAGAAGAAACG	GCCTCGTTGAGCTGGTGTTTGG	[38]
*sod*	TCGGAGACCTTGGTAATGT	CGCCTTCTCATGGATCAC	JQ776518.1
*cat*	TGAAGTTCTACACCGATGAG	CTGAGAGTGGACGAAGGA	XM_026238665.1
*gpx*	GAAGTGAACGGTGTGAACGC	GATCCCCCATCAAGGACACG	DQ983598.1
*ho-1*	GCAAACCAAGAGAAGCCACC	GGAAGTAGACGGGCTGAACC	KC758864
*nf-kb*	GCTCTGACTGCGGTCTTATAC	GCGCTTCATCGAGGATAGTT	[39]
*tgf-β*	GTTGGCGTAATAACCAGAAGG	AACAGAACAAGTTTGTACCGATAAG	[37]
*il-10*	AGTGAGACTGAAGGAGCTCCG	TGGCAGAATGGTGTCCAAGTA	[40]
*il-6*	CGGAGGGGCTTAACAGGATG	GCTGGCTCAGGAATGGGTAT	DQ861993.1
*il-8*	ATTGGTGAAGGAATGAGTCT	CCACAGATGACCTTGACAT	KC184490.1
*tnf-α*	CATTCCTACGGATGGCATTTACTT	CCTCAGGAATGTCAGTCTTGCAT	[37]
*il-1β*	GCGCTGCTCAACTTCATCTTG	GTGACACATTAAGCGGCTTCA C	[37]
*hif-1α*	CTGCCGATCAGTCTGTCTCC	TTTGTGGAGTCTGGACCACG	DQ306727.1
*epo*	CGAAGTGTCAGCATACCGGA	GCAGATGACGCACTTTTCCC	KC460317.1
*vegf*	ATCGAGCACACGTACATCCC	CCTTTGGCCTGCATTCACAC	NM_131408.3
*et1*	TAAAGCAGCGTCAGACAGGG	CTGCCAGCTTGTGTTTGCAT	NM_131519.1
*tf*	CCGAGAAGATGCACGCAAAG	TGTGCATGCCTTGACCAGAT	AF518747.1
*t* *fr* *-1*	CTTTGTCAACGAAGTGGCTGAAT	TACCAAAGAAAATGTGGCGGAAC	XM_052542523.1
*angpt1*	CCAAACCTCACCAAGCAAGC	GGATTACAGTCCAGCCTCCG	XM_059556208.1
*nos*	GGGGACCCTCCTGAAAATGG	TTCTGTCCTCAACGCTGGTG	AY644726.1

Note: *β-actin*, beta-actin; *sod*, superoxide dismutase; *cat*, catalase; *keap*1, Kelch-like ECH-associated protein1; *gpx*, glutathione peroxidase; *ho-1*, heme oxygenase-1; *nrf2*, nuclear factor erythroid 2-related factor 2; *tgf-β*, transforming growth factor-β; *il-10*, interleukin 10; *il-1β*, interleukin-1β; *tnf-α*, tumour necrosis factor-α; *nf-kb*, nuclear factor-kappa B; *il-8*, interleukin-8; *il-6*, interleukin 6; *vegf*, vascular endothelial growth factor; *hif-1*α, hypoxia-inducible factor-1α; *epo*, erythropoietin; *nos*, nitric oxide synthase; *et1*, endothelin; *angpt1*, angiopoietin-1; *tfr-1*, transferrin receptor protein 1; *tf*, transferrin.

**Table 3:** The ion concentrations of plasma and Na^+^/K^+^-ATPase activity of liver after hypoxia stress.

FPR Addition Level (%)	Na^+^ (mmol/L)	K^+^ (mmol/L)	Ca^+^ (mmol/L)	Cl^−^ (mmol/L)	Na^+^/K^+^-ATPase (U/mgprot)
0	82.58 ± 0.17 ^b^	17.31 ± 0.39 ^a^	1.42 ± 0.13 ^a^	70.08 ± 3.81	0.90 ± 0.04 ^b^
0.01	116.60 ± 14.98 ^ab^	16.94 ± 0.42 ^ab^	1.37 ± 0.03 ^a^	68.07 ± 0.88	1.55 ± 0.15 ^a^
0.02	191.10 ± 40.24 ^a^	15.39 ± 1.15 ^b^	0.87 ± 0.09 ^b^	66.77 ± 1.74	1.23 ± 0.19 ^ab^
0.03	128.78 ± 8.85 ^ab^	16.83 ± 0.43 ^ab^	0.80 ± 0.15 ^b^	65.64 ± 1.50	0.95 ± 0.30 ^b^
0.04	147.51 ± 19.90 ^ab^	16.61 ± 0.45 ^ab^	1.10 ± 0.14 ^ab^	64.17 ± 2.60	0.85 ± 0.21 ^b^

Note: Results are presented with the means ± SE. (n = 3). Results with different superscript letters (^a^, ^b^) represent significant differences (*p* < 0.05).

**Table 4:** The SR of gibel carp and number of mitochondria in gill after hypoxia stress.

FPR Supplementation Level (%)	Mitochondrial Number (Per Cell)	SR (%)
0	3.33 ± 1.86	23.81 ± 2.38 ^b^
0.01	6.00 ± 3.00	54.76 ± 2.38 ^a^
0.02	4.00 ± 2.08	52.38 ± 8.58 ^a^
0.03	4.33 ± 2.40	47.62 ± 2.38 ^a^
0.04	6.33 ± 1.76	47.62 ± 2.38 ^a^

Note: Results are presented with the means ± SE. (n = 3). Results with different superscript letters (^a^, ^b^) represent significant differences (*p* < 0.05).

## Data Availability

The data presented in this study are available on request from the corresponding author as the resultant data are contained within the article.

## Supplementary Materials

The following supporting information can be downloaded at: https://www.mdpi.com/article/10.3390/antiox14060738/s1, Table S1. Survival and death of fish were recorded in each tank after hypoxia stress.

## References

[1] Natarajan R., Fisher B.J., Fowler A.A. (2007). Hypoxia inducible factor-1 modulates hemin-induced IL-8 secretion in microvascular endothelium. Microvasc. Res..

[2] Rodrigues P.N.S., Pereira F.A. (2004). Effect of dietary iron overload on *Photobacterium damselae* ssp. *piscicida* pathogenicity in sea bass, *Dicentrarchus labrax* (L.). J. Fish Dis..

[3] Wu X.L., Li D.P., Lu J.M., Liu L., Yang Q.S., Tang R., Zhang X., Li L. (2023). Adaptation strategies of juvenile grass carp (*Ctenopharyngodon idella*) facing different dissolved oxygen concentrations in a recirculating aquaculture system. Water Biol. Secur..

[4] Sun H.J., Li J.J., Tang L.S., Yang Z. (2012). Responses of crucian carp *Carassius auratus* to long-term exposure to nitrite and low dissolved oxygen levels. Biochem. Syst. Ecol..

[5] Zhou D.S., Wang C.L., Zheng J.X., Zhao J.H., Wei S.S., Xiong Y.F., Limbu S.M., Kong Y.Q., Cao F., Ding Z.L. (2022). Dietary thiamine modulates carbohydrate metabolism, antioxidant status, and alleviates hypoxia stress in oriental river prawn *Macrobrachium nipponense* (de Haan). Fish Shellfish Immunol..

[6] Li M.X., Wang X.D., Qi C.L., Li E.E., Du Z.Y., Qin J.G., Chen L.Q. (2018). Metabolic response of Nile tilapia (*Oreochromis niloticus*) to acute and chronic hypoxia stress. Aquaculture.

[7] Dan X.M., Yan G.J., Zhang A.J., Cao Z.D., Fu S.J. (2014). Effects of stable and diel-cycling hypoxia on hypoxia tolerance, postprandial metabolic response, and growth performance in juvenile qingbo (*Spinibarbus sinensis*). Aquaculture.

[8] Shen Y.W., You W.W., Luo X., Lu Y., Huang M.Q., Ke C.H. (2023). An overview of the mechanisms underlying hypoxia tolerance differences in aquatic animals and their inspirations for aquaculture. Rev. Fish Biol. Fish..

[9] Xiao W.H. (2015). The hypoxia signaling pathway and hypoxic adaptation in fishes. Sci. China Life Sci..

[10] Mao X.J., Chen W.W., Long X.M., Pan X.M., Liu G.Q., Hu W.G., Gu D.C., Tan Q.S. (2024). Effect of dietary iron (Fe) level on growth performance and health status of largemouth bass (*Micropterus salmoides*). Aquaculture.

[11] Xiao K., Wang X., Wang M.M., Guo H.X., Liu W.B., Jiang G.Z. (2024). Metabolism, Antioxidant and Immunity in Acute and Chronic hypoxia Stress and the Improving Effect of Vitamin C in the Channel Catfish (*Ictalurus Punctatus*). Fish Physiol. Biochem..

[12] Lee J.H., Yoo Y.M., Lee B., Jeong S.H., Tran D.N., Jeung E.B. (2021). Melatonin mitigates the adverse effect of hypoxia during myocardial differentiation in mouse embryonic stem cells. J. Vet. Sci..

[13] Galeana-López J.A., Lizárraga-Velázquez C.E., Hernández C., Leyva-López N., Heredia J.B. (2021). Corn Husk Phenolics Modulate Hepatic Antioxidant Response in Nile Tilapia (*Oreochromis niloticus*) Exposed to Hypoxia. Molecules.

[14] Zhang Y.J., Zhao D., Xu J., Xu C.X., Dong C., Liu Q.W., Deng S.H., Zhao J., Zhang W., Chen X.J. (2013). Effects of Dietary Factors on the Pharmacokinetics of ^58^Fe-Labeled Hemin After Oral Administration in Normal Rats and the Iron-Deficient Rats. Biol. Trace Elem. Res..

[15] Ryter S.W. (2021). Significance of Heme and Heme Degradation in the Pathogenesis of Acute Lung and Inflammatory Disorders. Int. J. Mol. Sci..

[16] Foresti R., Goatly H., Green C.J., Motterlini R. (2001). Role of heme oxygenase-1 in hypoxia-reoxygenation: Requirement of substrate heme to promote cardioprotection. Am. J. Physiol. Heart Circ. Physiol..

[17] Tenhunen R., Marver H.S., Schmid R. (1968). The enzymatic conversion of heme to bilirubin by microsomal heme oxygenase. Proc. Natl. Acad. Sci. USA.

[18] Kumar D., Jena G.R., Ram M., Lingaraju M.C., Singh V., Prasad R., Kumawat S., Kant V., Gupta P., Tandan S.K. (2019). Hemin attenuated oxidative stress and inflammation to improve wound healing in diabetic rats. Naunyn-Schmiedeberg’s Arch. Pharmacol..

[19] Ndisang J.F., Tiwari S. (2015). Featured article: Induction of heme oxygenase with hemin improves pericardial adipocyte morphology and function in obese Zucker rats by enhancing proteins of regeneration. Exp. Biol. Med..

[20] Wang C.H., Zhang Y.J., Han L.L., Guo L., Zhong H., Wang J.W. (2017). Hemin ameliorates influenza pneumonia by attenuating lung injury and regulating the immune response. Int. J. Antimicrob. Agents.

[21] Lee J.M., Lee W.H., Kay H.Y., Kim E., Moon A., Kim S.G. (2012). Hemin, an iron-binding porphyrin, inhibits HIF-1α induction through its binding with heat shock protein 90. Int. J. Cancer.

[22] He J., Yu Y., Qin X.W., Zeng R.Y., Wang Y.Y., Li Z.M., Mi S., Weng S.P., Guo C.J., He J.G. (2019). Identification and functional analysis of the Mandarin fish (*Siniperca chuatsi*) hypoxia-inducible factor-1α involved in the immune response. Fish Shellfish Immunol..

[23] Lin Y., Miao L.H., Zhang W.X., Pan W.J., Liang H.L., Ge X.P., Xu Y.S., Liu B., Ren M.C., Zhou Q.L. (2018). Effect of nitrite exposure on oxygen-carrying capacity and gene expression of NF-κB/HIF-1α pathway in gill of bighead carp (*Aristichthys nobilis*). Aquacult. Int..

[24] Huang Z.H., Guan W.L., Wei X.B., Chen R.C., Lyu X.M., Zheng G.H., Mao L.C. (2023). Examination of the role of hypoxia-inducible factor-1α (HIF-1α) in preventing hemocyte apoptosis in whiteleg shrimp (*Litopenaeus vannamei*). Aquaculture.

[25] Aschner M., Skalny A.V., Lu R., Santamaria A., Zhou J.C., Ke T., Karganov M.Y., Tsatsakis A., Golokhvast K.S., Bowman A.B. (2023). The role of hypoxia-inducible factor 1 alpha (HIF-1α) modulation in heavy metal toxicity. Arch. Toxicol..

[26] Ni Q.G., Wang D., Xie L.L., Ge H.X., Dong Z.G. (2022). Unravelling the characterization of hypoxia-inducible factor-1α (HIF-1α) and antioxidant enzymes in clam Cyclina sinensis in response to hypoxia. Aquacult. Res..

[27] Hänze J., Eul B.G., Savai R., Krick S., Goyal P., Grimminger F., Seeger W., Rose F. (2003). RNA interference for HIF-1α inhibits its downstream signalling and affects cellular proliferation. Biochem. Biophys. Res. Commun..

[28] Metzen E., Zhou J., Jelkmann W., Fandrey J., Brüne B. (2003). Nitric Oxide Impairs Normoxic Degradation of HIF-1α by Inhibition of Prolyl Hydroxylases. Mol. Biol. Cell.

[29] Rahman M.S., Thomas P. (2007). Molecular cloning, characterization and expression of two hypoxia-inducible factor alpha subunits, HIF-1α and HIF-2α, in a hypoxia-tolerant marine teleost, Atlantic croaker (*Micropogonias undulatus*). Gene.

[30] Mladineo I., Block B.A. (2009). Expression of Hsp70, Na^+^/K^+^ ATP-ase, HIF-1α, IL-1β and TNF-α in captive Pacific bluefin tuna (*Thunnus orientalis*) after chronic warm and cold exposure. J. Exp. Mar. Biol. Ecol..

[31] Li H.T., Lu L., Wu M., Xiong X.Q., Luo L., Ma Y.T., Liu Y. (2020). The effects of dietary extract of mulberry leaf on growth performance, hypoxia-reoxygenation stress and biochemical parameters in various organs of fish. Aquacult. Rep..

[32] Wu L.Y., Xu W.J., Li H.Y., Dong B., Geng H.C., Jin J.Y., Han D., Liu H.K., Zhu X.M., Yang Y.X. (2022). Vitamin C Attenuates Oxidative Stress, Inflammation, and Apoptosis Induced by Acute Hypoxia through the Nrf2/Keap1 Signaling Pathway in Gibel Carp (*Carassius gibelio*). Antioxidants.

[33] Liu H., Zhu X., Yang Y., Han D., Jin J., Xie S. (2016). Effect of substitution of dietary fishmeal by soya bean meal on different sizes of gibel carp (*Carassius auratus gibelio*): Nutrient digestibility, growth performance, body composition and morphometry. Aquacult. Nutr..

[34] Wang B., Mao H., Zhao J., Liu Y., Wang Y., Du X. (2023). Influences of oxygen and temperature interaction on the antibacterial activity, antioxidant activity, serum biochemical indices, blood indices and growth performance of crucian carp. Peerj.

[35] Xing M., Rong Z., Zhao X., Gao X., Hou Z., Zhang L., Khor W., Xu Y., Chen L., Wu C. (2025). Transcriptome analysis reveals hypoxic response key genes and modules as well as adaptive mechanism of crucian carp (*Carassius auratus*) gill under hypoxic stress. Front. Immunol..

[36] Wang K., Zhang L., Liang H.L., Ren M.C., Mi H.F., Huang D.Y., Gu J.Z. (2024). Effects of Dietary Ferroporphyrin Supplementation on Growth Performance, Antioxidant Capacity, Immune Response, and Oxygen-Carrying Capacity in Gibel Carp (*Carassius Auratus Gibelio*). Animals.

[37] Yang K.C., Qi X.Z., He M.S., Song K.G., Luo F., Qu X.Y., Wang G.X., Ling F. (2020). Dietary supplementation of salidroside increases immune response and disease resistance of Gibel Carp (*Carassius auratus*) against *Aeromonas hydrophila*. Fish Shellfish Immunol..

[38] Sun L., Wang Q., Wang R., Sun K., Li S., Lin G., Lei P., Xu H. (2022). Effect of dietary poly-γ-glutamic acid on growth, digestive enzyme activity, antioxidant capacity, and TOR pathway gene expression of gibel carp (*Carassius auratus gibelio*). Aquacult. Rep..

[39] Gu Y.P., Chen K., Xi B.W., Xie J., Bing X.W. (2022). Protective effects of paeonol against lipopolysaccharide-induced liver oxidative stress and inflammation in gibel carp (*Carassius auratus gibelio*). Comp. Biochem. Physiol. C.

[40] Khieokhajonkhet A., Suwannalers P., Aeksiri N., Ratanasut K., Chitmanat C., Inyawilert W., Phromkunthong W., Kaneko G. (2023). Effects of dietary red pepper extracts on growth, hematology, pigmentation, disease resistance, and growth- and immune-related gene expressions of goldfish (*Carassius auratus*). Anim. Feed Sci. Technol..

[41] Obirikorang K.A., Acheampong J.N., Duodu C.P., Skov P.V. (2020). Growth, metabolism and respiration in Nile tilapia (Oreochromis niloticus) exposed to chronic or periodic hypoxia. Comp. Biochem. Phys. A.

[42] Qin F.J., Shi M.M., Yuan H.X., Yuan L.X., Lu W.H., Zhang J., Tong J., Song X.H. (2016). Dietary nano-selenium relieves hypoxia stress and, improves immunity and disease resistance in the Chinese mitten crab (*Eriocheir sinensis*). Fish Shellfish Immunol..

[43] Zhang L.M., Zhang L., Liang H.L., Huang D.Y., Ren M.C. (2024). Effects of Taurine and Vitamin C on the Improvement of Antioxidant Capacity, Immunity and Hypoxia Tolerance in Gibel Carp (*Carrassius auratus gibeilo*). Antioxidants.

[44] Ma Q., Xu H.G., Wei Y.L., Liang M.Q. (2024). Effects of acute hypoxia on nutrient metabolism and physiological function in turbot, *Scophthalmus maximus*. Fish Physiol. Biochem..

[45] Shuang L., Chen S.L., Ren C., Su X.L., Xu X.N., Zheng G.D., Zou S.M. (2023). Effects of hypoxia and reoxygenation on oxidative stress, histological structure, and apoptosis in a new hypoxia-tolerant variety of blunt snout bream (*Megalobrama amblycephala*). Comp. Biochem. Phys. A.

[46] Zhao Y.J., Jiang X.Y., Kong X.H., Di G.L., Nie G.X., Li X.J. (2017). Effects of hypoxia on lysozyme activity and antioxidant defences in the kidney and spleen of *Carassius auratus*. Aquac. Res..

[47] Shi Q.C., Yu C.Q., Zhu D.S., Li S.K., Wen X.B. (2020). Effects of dietary Sargassum horneri on resisting hypoxia stress, which changes blood biochemistry, antioxidant status, and hepatic HSP mRNA expressions of juvenile black sea bream *Acanthopagrus schlegelii*. J. Appl. Phycol..

[48] Yu H., Ge X.P., Zhang L., Chen X.R., Ren M.C., Liang H.L. (2023). Transcriptome analysis reveals the feeding response and oxidative stress in juvenile *Micropterus salmoides* fed a low-fish-meal diet with enzyme-hydrolysed intestinal mucosa proteinsubstitution. Aquaculture.

[49] Cooper R.U., Clough L.M., Farwell M.A., West T.L. (2002). Hypoxia-induced metabolic and antioxidant enzymatic activities in the estuarine fish *Leiostomus xanthurus*. J. Exp. Mar. Biol. Ecol..

[50] Yang S., Yan T., Wu H., Xiao Q., Fu H.M., Luo J., Zhou J., Zhao L.L., Wang Y., Yang S.Y. (2017). Acute hypoxic stress: Effect on blood parameters, antioxidant enzymes, and expression of HIF-1alpha and GLUT-1 genes in largemouth bass (*Micropterus salmoides*). Fish Shellfish Immunol..

[51] Wu L.Y., Li H.Y., Xu W.J., Dong B., Geng H.C., Jin J.Y., Han D., Liu H.K., Zhu X.M., Yang Y.X. (2022). Emodin alleviates acute hypoxia-induced apoptosis in gibel carp (*Carassius gibeli*o) by upregulating autophagy through modulation of the AMPK/mTOR pathway. Aquaculture.

[52] Liang H.L., Mokrani A., Ji K., Ge X.P., Ren M.C., Xie J., Liu B., Xi B.W., Zhou Q.L. (2018). Dietary leucine modulates growth performance, Nrf2 antioxidant signaling pathway and immune response of juvenile blunt snout bream (*Megalobrama amblycephala*). Fish Shellfish Immunol..

[53] Fouad A.A., Qureshi H.A., Al-Sultan A.I., Yacoubi M.T., Ali A.A. (2009). Protective effect of hemin against cadmium-induced testicular damage in rats. Toxicology.

[54] Wu Y., Guo M.T., Hua X.J., Duan K.X., Lian G.H., Sun L., Tang L.J., Xu Y.G., Liu M., Li Y.J. (2017). The role of infectious hematopoietic necrosis virus (IHNV) proteins in the modulation of NF-κB pathway during IHNV infection. Fish Shellfish Immunol..

[55] Nam S.E., Haque M.N., Shin Y.K., Park H.S., Rhee J.S. (2020). Constant and intermittent hypoxia modulates immunity, oxidative status, and blood components of red seabream and increases its susceptibility to the acute toxicity of red tide dinoflagellate. Fish Shellfish Immunol..

[56] Yang Z.T., Wang L., Wong S.M., Yue G.H. (2020). The *HIF1* an gene and its association with hypoxia tolerance in the Asian seabass. Gene.

[57] Chen X., Feng W.R., Yan F.Y., Li W.J., Xu P., Tang Y.K. (2023). Alteration of antioxidant status, glucose metabolism, and hypoxia signal pathway in Eirocheir sinensis after acute hypoxic stress and reoxygenation. Comp. Biochem. Phys. C.

[58] Nie H.T., Wang H.M., Jiang K.Y., Yan X.W. (2020). Transcriptome analysis reveals differential immune related genes expression in *Ruditapes philippinarum* under hypoxia stress: Potential HIF and NF-κB crosstalk in immune responses in clam. BMC Genom..

[59] Zhang T., Guo S., Zhu X.Y., Qiu J.X., Deng G.Z., Qiu C.W. (2020). Alpinetin inhibits breast cancer growth by ROS/NF-κB/HIF-1α axis. J. Cell. Mol. Med..

[60] Shi X.W., Gao F., Zhao X.L., Pei C., Zhu L., Zhang J., Li C., Li L., Kong X.H. (2023). Role of HIF in Fish Inflammation. Fish Shellfish Immunol..

[61] Ferla K.L., Reimann C., Jelkmann W., Hellwig-Bürgel T. (2002). Inhibition of erythropoietin gene expression signaling involves the transcription factors GATA-2 and NF-κB. FASEB J..

[62] Whitehouse L.M., Manzon R.G. (2019). Hypoxia alters the expression of hif-1a mRNA and downstream HIF-1 response genes in embryonic and larval lake whitefish (*Coregonus clupeaformis*). Comp. Biochem. Phys. A.

[63] Biswas S., Tapryal N., Mukherjee R., Kumar R., Mukhopadhyay C.K. (2013). Insulin promotes iron uptake in human hepatic cell by regulating transferrin receptor-1 transcription mediated by hypoxia inducible factor-1. BBA-Mol. Basis Dis..

[64] Zheng C., Zhao Q.Q., Li E.C., Zhao D.X., Sun S.M. (2022). Role of hypoxia in the behaviour, physiology, immunity and response mechanisms of crustaceans: A review. Rev. Aquac..

[65] Falfushynska H., Sokolova I.M. (2023). Intermittent hypoxia differentially affects metabolic and oxidative stress responses in two species of cyprinid fish. Biol. Open.

[66] Filice M., Gattuso A., Imbrogno S., Mazza R., Amelio D., Caferro A., Agnisola C., Icardo J.M., Cerra M.C. (2024). Functional, structural, and molecular remodelling of the goldfish (*Carassius auratus*) heart under moderate hypoxia. Fish Physiol. Biochem..

[67] Yu X.X., Zhang Y.R., Li S.S., Zheng G.D., Zou S.M. (2023). Effects of hypoxia on the gill morphological structure, apoptosis and hypoxia-related gene expression in blunt snout bream (*Megalobrama amblycephala*). Fish Physiol. Biochem..

[68] Steffen J.B.M., Sokolov E.P., Bock C., Sokolova I.M. (2023). Combined effects of salinity and intermittent hypoxia on mitochondrial capacity and reactive oxygen species efflux in the Pacific oyster, *Crassostrea gigas*. J. Exp. Biol..

[69] Song Y.M., Wu M.Y., Pang Y.Y., Song X.Z., Shi A., Shi X.L., Niu C., Cheng Y.X., Yang X.Z. (2021). Effects of melatonin feed on the changes of hemolymph immune parameters, antioxidant capacity, and mitochondrial functions in Chinese mitten crab (*Eriocheir sinensis*) caused by acute hypoxia. Aquaculture.

[70] Hao T.S., Yu J.L., Wu Z.D., Jiang J., Gong L.L., Wang B.J., Guo H.Z., Zhao H.B., Lu B., Engelender S. (2023). Hypoxia-reprogramed megamitochondrion contacts and engulfs lysosome to mediate mitochondrial self-digestion. Nat. Commun..

[71] Shen H.T., Liang P., Qiu S.H., Zhang B., Wang Y.L., Lv P. (2016). The role of Na^+^, K^+^-ATPase in the hypoxic vasoconstriction in isolated rat basilar artery. Vasc. Pharmacol..

[72] Huang D.Y., Zhu J., Xu G.C., Zhang L., Chen X.R., Wang Y.L., Ren M.C., Liang H.L. (2023). Sodium chloride alleviates oxidative stress and physiological responses induced by extreme winter cold in genetically improved farmed tilapia (GIFT; *Oreochromis niloticus*). Sci. Total Environ..

[73] Dada L.A., Chandel N.S., Ridge K.M., Pedemonte C., Bertorello A.M., Sznajder J.I. (2003). Hypoxia-induced endocytosis of Na,K-ATPase in alveolar epithelial cells is mediated by mitochondrial reactive oxygen species and PKC-ζ. J. Clin. Investig..

[74] Comellas A.P., Dada L.A., Lecuona E., Pesce L.M., Chandel N.S., Quesada N., Budinger G.R.S., Strous G.J., Ciechanover A., Sznajder J.I. (2006). Hypoxia-Mediated Degradation of Na,K-ATPase via Mitochondrial Reactive Oxygen Species and the Ubiquitin-Conjugating System. Circ. Res..

