# Supplementary material for: Enhancement of Hypoxia Tolerance of Gibel Carp (Carassius auratus gibelio) via a Ferroporphyrin-Rich Diet
Source: Antioxidants (Basel). 2025 Jun 16;14(6):738. doi: 10.3390/antiox14060738 (PMC12189492; doi:10.3390/antiox14060738)
Supplement: Supplementary file 1 [file antioxidants-14-00738-s001.zip › antioxidants-3612097-supplementary.pdf]

Supplementary materials

Table S1 Survival and death of fish were recorded in each tank after hypoxia stress

FPR addition level (%)	Total fish	Survival fish	Death fish	Survival rate (SR, %)
0	14	3	11	21.43
0	14	4	10	28.57
0	14	3	11	21.43
0.01	14	8	6	57.14
0.01	14	7	7	50.00
0.01	14	8	6	57.14
0.02	14	5	9	35.71
0.02	14	9	5	64.29
0.02	14	8	6	57.14
0.03	14	7	7	50.00
0.03	14	7	7	50.00
0.03	14	6	8	42.86
0.04	14	7	6	50.00
0.04	14	7	7	50.00
0.04	14	6	8	42.86

Note: Survival rate (SR, %) = 100 × (survival fish number/total fish).
